# Supplementary figures and images for: A non-bactericidal cathelicidin provides prophylactic efficacy against bacterial infection by driving phagocyte influx
Source: eLife. 2022 Feb 23;11:e72849. doi: 10.7554/eLife.72849 (PMC8865851; doi:10.7554/eLife.72849)

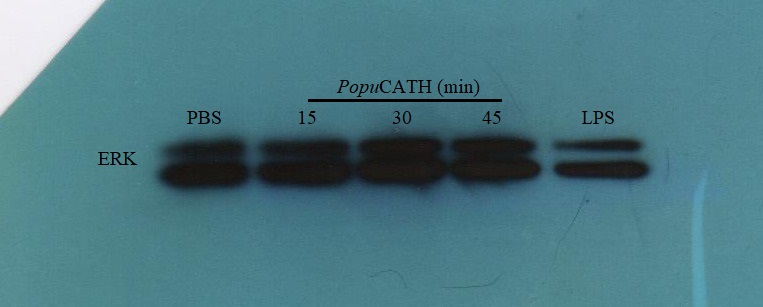

Supplement: Figure 9—source data 1. [file elife-72849-fig9-data1.zip › Figure 9-source data 1/Figures with the uncropped blots with the relevant bands clearly labelled/ERK.jpg]

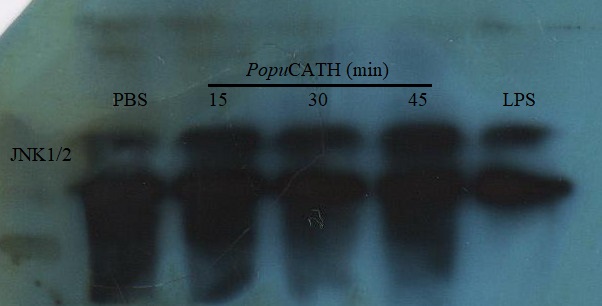

Supplement: Figure 9—source data 1. [file elife-72849-fig9-data1.zip › Figure 9-source data 1/Figures with the uncropped blots with the relevant bands clearly labelled/JNK.jpg]

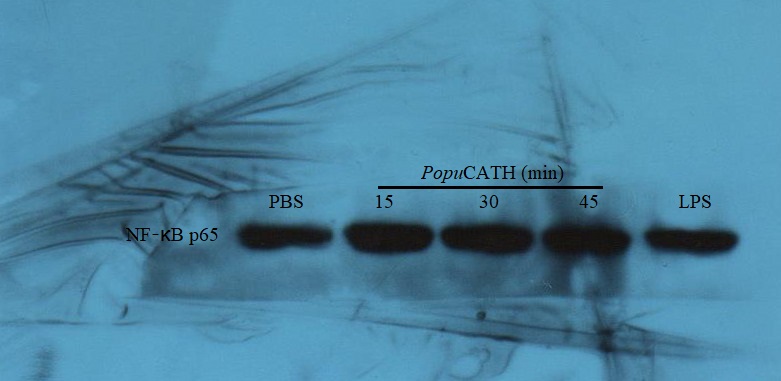

Supplement: Figure 9—source data 1. [file elife-72849-fig9-data1.zip › Figure 9-source data 1/Figures with the uncropped blots with the relevant bands clearly labelled/NF-κB p65.jpg]

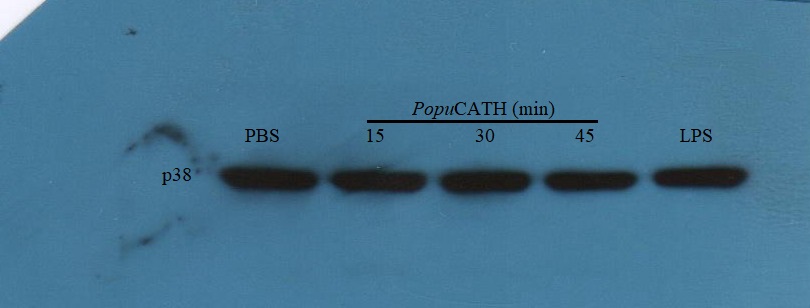

Supplement: Figure 9—source data 1. [file elife-72849-fig9-data1.zip › Figure 9-source data 1/Figures with the uncropped blots with the relevant bands clearly labelled/p38.jpg]

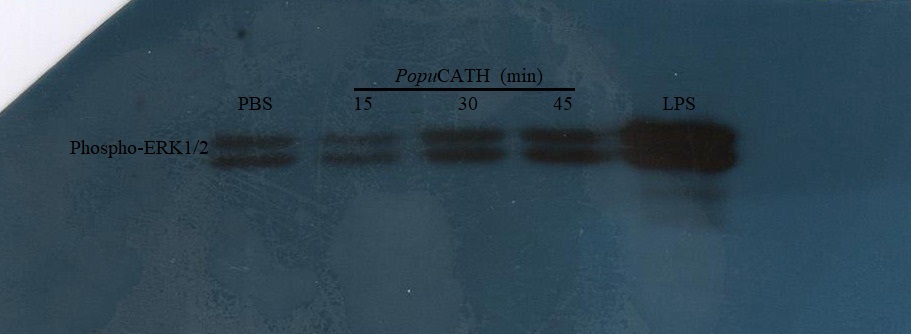

Supplement: Figure 9—source data 1. [file elife-72849-fig9-data1.zip › Figure 9-source data 1/Figures with the uncropped blots with the relevant bands clearly labelled/Phospho-ERK.jpg]

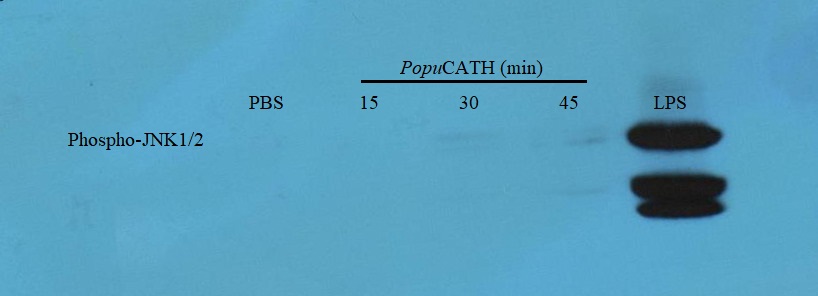

Supplement: Figure 9—source data 1. [file elife-72849-fig9-data1.zip › Figure 9-source data 1/Figures with the uncropped blots with the relevant bands clearly labelled/Phospho-JNK.jpg]

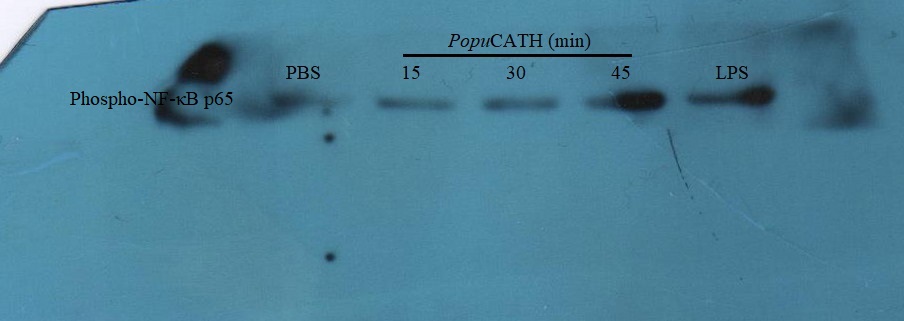

Supplement: Figure 9—source data 1. [file elife-72849-fig9-data1.zip › Figure 9-source data 1/Figures with the uncropped blots with the relevant bands clearly labelled/Phospho-NF-κB p65.jpg]

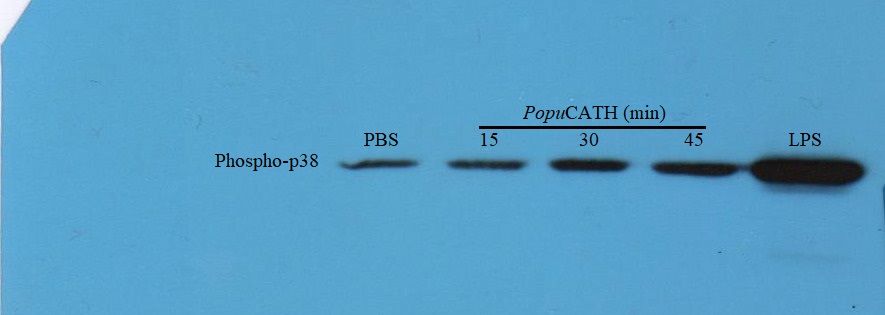

Supplement: Figure 9—source data 1. [file elife-72849-fig9-data1.zip › Figure 9-source data 1/Figures with the uncropped blots with the relevant bands clearly labelled/Phospho-p38.jpg]

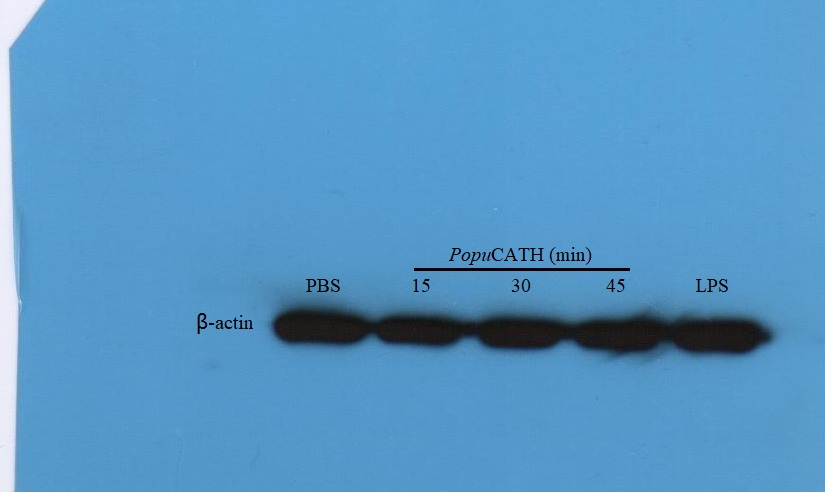

Supplement: Figure 9—source data 1. [file elife-72849-fig9-data1.zip › Figure 9-source data 1/Figures with the uncropped blots with the relevant bands clearly labelled/β-actin.jpg]

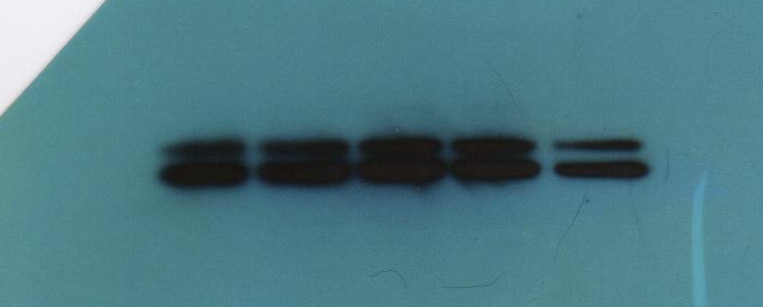

Supplement: Figure 9—source data 1. [file elife-72849-fig9-data1.zip › Figure 9-source data 1/Original files of the full raw unedited blots/ERK.jpg]

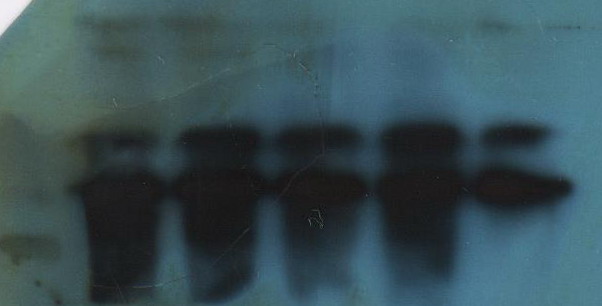

Supplement: Figure 9—source data 1. [file elife-72849-fig9-data1.zip › Figure 9-source data 1/Original files of the full raw unedited blots/JNK.jpg]

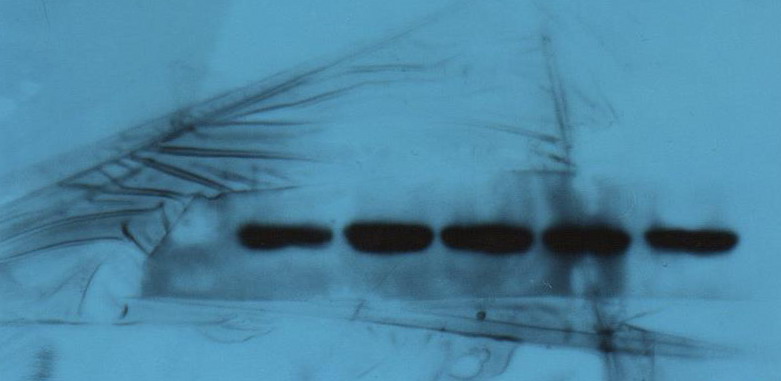

Supplement: Figure 9—source data 1. [file elife-72849-fig9-data1.zip › Figure 9-source data 1/Original files of the full raw unedited blots/NF-κB p65.jpg]

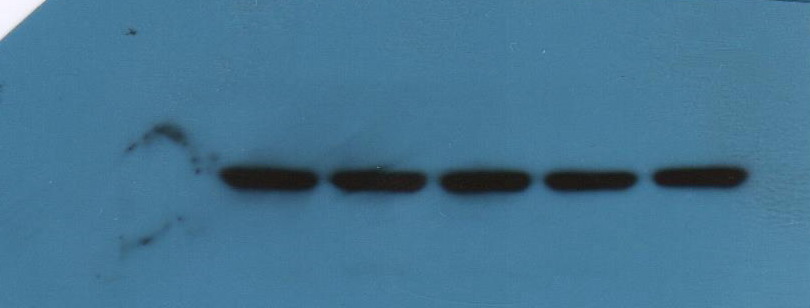

Supplement: Figure 9—source data 1. [file elife-72849-fig9-data1.zip › Figure 9-source data 1/Original files of the full raw unedited blots/p38.jpg]

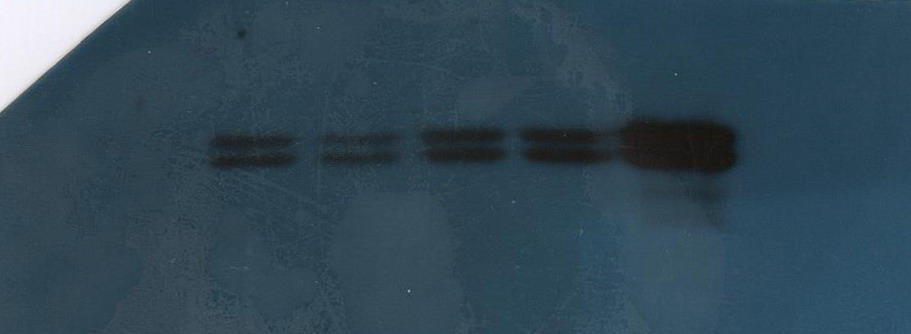

Supplement: Figure 9—source data 1. [file elife-72849-fig9-data1.zip › Figure 9-source data 1/Original files of the full raw unedited blots/Phospho-ERK.jpg]

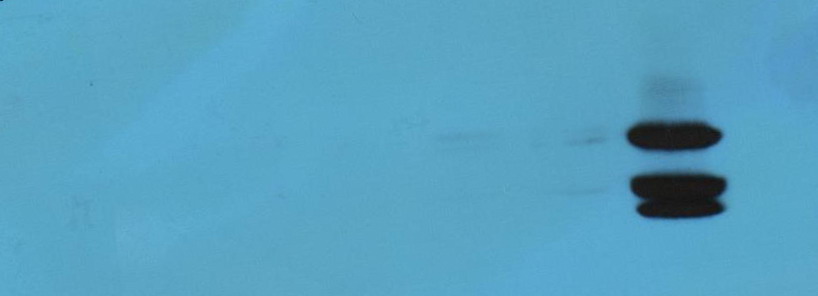

Supplement: Figure 9—source data 1. [file elife-72849-fig9-data1.zip › Figure 9-source data 1/Original files of the full raw unedited blots/Phospho-JNK.jpg]

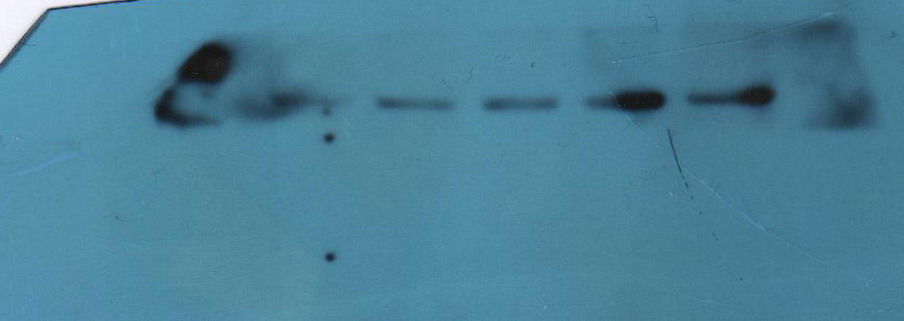

Supplement: Figure 9—source data 1. [file elife-72849-fig9-data1.zip › Figure 9-source data 1/Original files of the full raw unedited blots/Phospho-NF-κB p65.jpg]

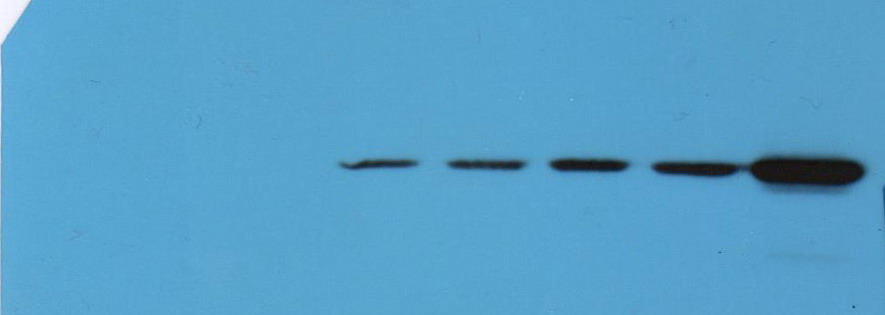

Supplement: Figure 9—source data 1. [file elife-72849-fig9-data1.zip › Figure 9-source data 1/Original files of the full raw unedited blots/Phospho-p38.jpg]

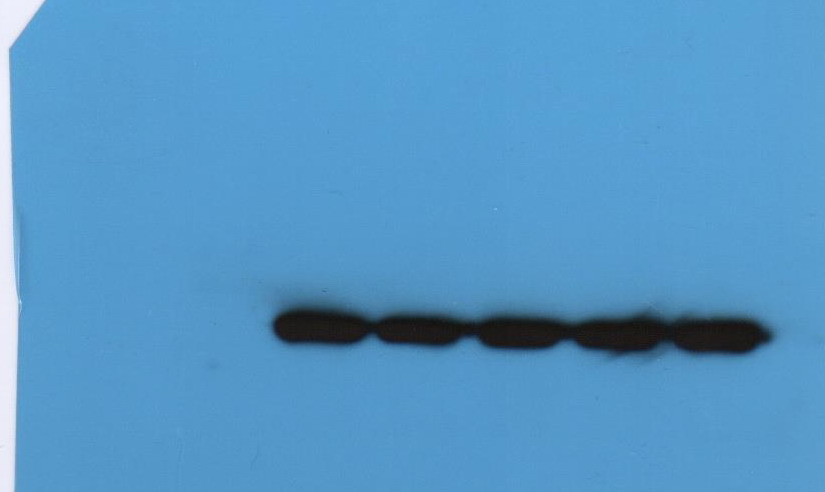

Supplement: Figure 9—source data 1. [file elife-72849-fig9-data1.zip › Figure 9-source data 1/Original files of the full raw unedited blots/β-actin.jpg]
