# Supplementary material for: A non-bactericidal cathelicidin provides prophylactic efficacy against bacterial infection by driving phagocyte influx
Source: eLife. 2022 Feb 23;11:e72849. doi: 10.7554/eLife.72849 (PMC8865851; doi:10.7554/eLife.72849)
Supplement: Supplementary file 1. [file elife-72849-supp1.docx]

**Supplementary file 1. Physico-chemical parameters of *Popu*CATH.**

| Total amino acid residues | Polar residues | Non-polar residues | Rich residue | Net charge | Theoretical  pI*^a^* | Theoretical MW*^b^* (Da) | Observed MW (Da) |
| --- | --- | --- | --- | --- | --- | --- | --- |
|  |  |  | Gly |  |  |  |  |
| 46 | 41 | 5 | 21 | +10 | 12.60 | 4296.54 | 4295.90 |

*^a^*isoelectric point, *^b^*molecular weight
