# Supplementary material for: A non-bactericidal cathelicidin provides prophylactic efficacy against bacterial infection by driving phagocyte influx
Source: eLife. 2022 Feb 23;11:e72849. doi: 10.7554/eLife.72849 (PMC8865851; doi:10.7554/eLife.72849)
Supplement: Supplementary file 2. [file elife-72849-supp2.docx]

**Supplementary file 2. Secondary structural components of *Popu*CATH in aqueous solution and membrane-mimetic solution.**

| Solution | Helix (%)^a^ | Beta (%)^a^ | Turn (%)^a^ | Random (%)^a^ |
| --- | --- | --- | --- | --- |
| H_2_O | 0.0 | 15.8 | 27.2 | 57.0 |
| TFE/H_2_O |  |  |  |  |
| 3:7 | 0.0 | 36.6 | 15.0 | 48.4 |
| 5:5 | 1.9 | 46.4 | 4.3 | 47.4 |
| 7:3 | 3.2 | 39.0 | 19.1 | 38.8 |
| 9:1 | 5.1 | 53.0 | 0.0 | 41.9 |
| SDS (mM) |  |  |  |  |
| 5 | 0.0 | 3.2 | 25.6 | 71.2 |
| 10 | 0.0 | 29.1 | 22.6 | 48.3 |
| 20 | 0.0 | 47.3 | 20.9 | 31.8 |
| 40 | 0.0 | 33.2 | 22.9 | 43.9 |

^a^Jasco-810 software was used to deconvolute CD spectra into fractional contents and these data are the average value of three scans.
