# Supplementary material for: A non-bactericidal cathelicidin provides prophylactic efficacy against bacterial infection by driving phagocyte influx
Source: eLife. 2022 Feb 23;11:e72849. doi: 10.7554/eLife.72849 (PMC8865851; doi:10.7554/eLife.72849)
Supplement: Supplementary file 3. [file elife-72849-supp3.docx]

**Supplementary file 3. MIC values of *Popu*CATH against Gram-negative bacteria, Gram-positive bacteria, fungi, and aquatic bacteria.**

| Microorganisms | MIC*^a^* (μg/ml) | |
| --- | --- | --- |
|  | *Popu*CATH | PY*^b^* |
| Gram-negative bacteria |  |  |
| *Escherichia coli* ATCC25922 | >200 | 4.69 |
| *Escherichia coli* ATCC35218 | >200 | 9.38 |
| *Escherichia coli* 13A10022 (CI*^c^*) | >200 | 18.75 |
| *Escherichia coli* 08A852 (CI) | >200 | 4.69 |
| *Escherichia coli* 08A866 (CI) | >200 | 9.38 |
| *Escherichia coli* 13U (CI) | >200 | 4.69 |
| *Pseudomonas aeruginosa* ATCC27853 | >200 | 18.75 |
| *Pseudomonas aeruginosa* 08031205 (CI) | >200 | 9.38 |
| *Pseudomonas aeruginosa* 08031014 (CI) | >200 | 37.5 |
| *Acinetobacter baumannii* ATCC19606 | >200 | 75 |
| *Acinetobacter junii* 13A15578 (CI) | >200 | 37.5 |
| *Merulius morganii* 13A15448 (CI) | >200 | ND*^d^* |
| *Salmonella paratyphi-*A | >200 | ND |
| *Klebsiella pneumoniae* 0813343 (CI) | >200 | 37.5 |
| *Klebsiella pneumoniae* 08040202 (CI) | >200 | ND |
| *Klebsiella pneumoniae* 08040724 (CI) | >200 | ND |
| *Alcaligenes faecalis* 13A154089 (CI) | >200 | ND |
| *Shigella castellani* | >200 | ND |
|  |  |  |
| Gram-positive bacteria |  |  |
| *Staphylococcus aureus* ATCC25923 | >200 | 37.5 |
| *Staphylococcus aureus* 08A875 (CI) | >200 | 18.75 |
| *Staphylococcus aureus* 08A865 (CI) | >200 | 9.38 |
| *Streptococcus pyogenes* ATCC19615 | >200 | 37.5 |
| *Staphylococcus haemolyticus* 13A13770 (CI) | >200 | ND |
| *Staphylococcus epidermidis* 13A13730 (CI) | >200 | ND |
| *Bacillus subtilis subtilis* ATCC 6633 | >200 | 150 |
| *[Enterococcus](http://www.sogou.com/link?url=B7ss7T7Ay4n1v-RDViKyPWuDQ7vn3ukUbhhp9J6i36M.&query=E.faecalis) faecalis* ATCC29212 | >200 | ND |
| [*Enterococcus*](http://www.sogou.com/link?url=B7ss7T7Ay4n1v-RDViKyPWuDQ7vn3ukUbhhp9J6i36M.&query=E.faecalis) *faecalis* 13U1964 (CI) | >200 | ND |
| [*Enterococcus*](http://www.sogou.com/link?url=B7ss7T7Ay4n1v-RDViKyPWuDQ7vn3ukUbhhp9J6i36M.&query=E.faecalis) *faecalis* 14U00168 | >200 | ND |
|  |  |  |
| Fungi |  |  |
| *Candida albicans* ATCC2002 | >200 | 4.69 |
| *Candida albicans* ATCC14053 | >200 | 37.5 |
| *Candida albicans* 08022710 (CI) | >200 | 18.75 |
| *Candida albicans* 08030401 (CI) | >200 | 18.75 |
| *Candida albicans* 08A802 (CI) | >200 | 9.38 |
| *Candida albicans* 08030804 (CI) | >200 | 4.69 |
| *Candida albicans* 08030809 (CI) | >200 | 4.69 |
|  |  |  |
| Aquatic bacteria |  |  |
| *Aeromonas hydrophila* | >200 | 9.38 |
| *Aeromonas veronii* | >200 | ND |
| *Vibrio harveyi* | >200 | 18.75 |
| *Vibrio cholerae* | >200 | ND |
| *Vibrio anguillarum* | >200 | ND |

*^a^*MIC, minimal inhibitory concentrations represent mean values of three independent experiments performed in duplicate. *^b^*PY, cathelicidin-PY, an amphibian cathelicidin from the frog *Paa yunnanensis* with direct antimicrobial activity was used as positive control. *^c^*CI, clinically isolated strain. *^d^*ND, not determined.
