# Supplementary material for: A non-bactericidal cathelicidin provides prophylactic efficacy against bacterial infection by driving phagocyte influx
Source: eLife. 2022 Feb 23;11:e72849. doi: 10.7554/eLife.72849 (PMC8865851; doi:10.7554/eLife.72849)
Supplement: Supplementary file 4. [file elife-72849-supp4.pdf]

AZENTA
LIFE SCIENCES

Cell Line Authentication Report

Cell Line Authentication Report

Customer: Jing Wang

Quotation Number: 80-824993409_R2

Completion Date: 12/16/2021

1. **Sample ID: THP-1**
2. **Original Material: Cell pellet**
3. **Methods:**

1).Genomic DNA was extracted from the cell pellets provided by the customer.

2).Samples, together with positive and negative control were amplified using GenePrint 10 System (Promega).

3). Amplified products were processed using the ABI3730xl Genetic Analyzer.

4). Data were analyzed using GeneMapper4.0 software and then compared with the ATCC, DSMZ, JCRB and RIKEN databases for reference matching.

4. **Results:**

1) **10 Loci STR Profile：**

Genetic Site	Cell Bank information		Customer sample	
(Locus)	THP-1		THP-1	
Amelogenin	X	Y	X	Y
CSF1PO	11	13	11	13
D13S317	13	13	13	13
D16S539	11	12	11	12
D5S818	11	12	11	12
D7S820	10	10	10	10
THO1	8	9.3	8	9.3
TPOX	8	11	8	11
vWA	16	16	16	16
D21S11			30	31.2
Percent match between the sample and the database profile：100%				

Summary:

Your cell line is considered to be “identical” to the reference cell line in the Cell Bank STR database, as the STR profile yields a 100% match.

Notes：

1. P=100% x (2xM)/N; M=18, N=36 P=100% x (2x18)/36=100%

M: number of the matching peaks; N: number of all peaks

2. Based on the ANSI Standard, cell lines with ≥80% match are considered to be related; i.e., derived from a common ancestry. Cell lines with between a 55% to 80% match require further profiling for authentication of relatedness.

3. The short tandem repeat (STR) profile generated by GENEWIZ Inc. is indicative only of the sample sent to GENEWIZ Inc. at the time it was sent. This data and analysis are for research use only.

2) Electrophoretogram

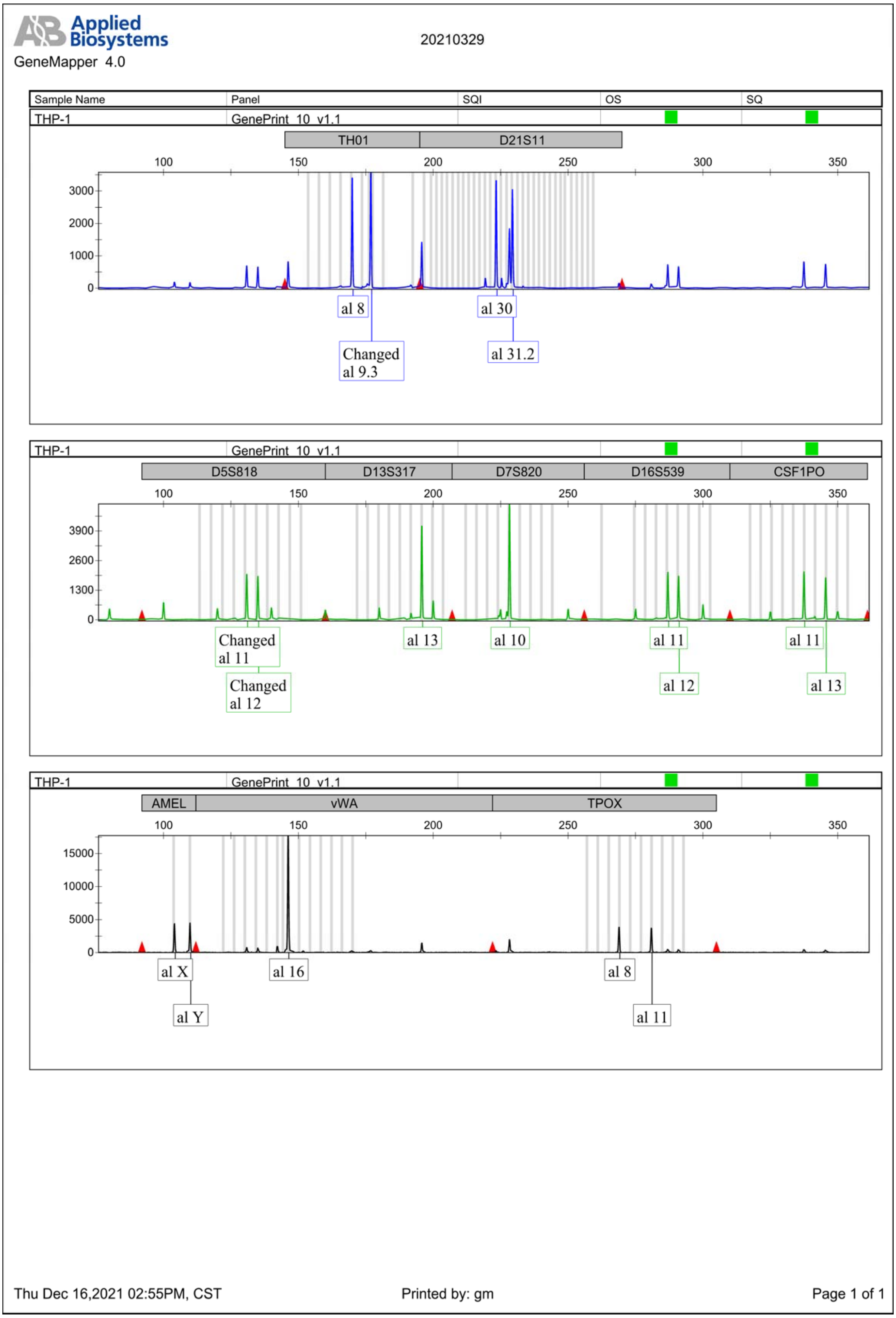

Note：Raw data in appendix
