# Supplementary material for: A non-bactericidal cathelicidin provides prophylactic efficacy against bacterial infection by driving phagocyte influx
Source: eLife. 2022 Feb 23;11:e72849. doi: 10.7554/eLife.72849 (PMC8865851; doi:10.7554/eLife.72849)
Supplement: Supplementary file 5. [file elife-72849-supp5.docx]

**Supplementary file 5. Primers for qPCR.**

| **Primer** | **Forward (5’→3’)** | **Reverse (5’→3’)** |
| --- | --- | --- |
| *Ccl1* | GCAAGAGCATGCTTACGGTCT | TAGTTGAGGCGCAGCTTTCT |
| *Ccl2* | GCTGTAGTTTTTGTCACCAAGC | GTGCTGAAGACCTTAGGGCA |
| *Ccl3* | CCATATGGAGCTGACACCCC | TCAGGAAAATGACACCTGGCT |
| *Ccl4* | CCAGCTGTGGTATTCCTGACC | AGCAAGGACGCTTCTCAGTG |
| *Ccl5* | GACAGCACATGCATCTCCCA | GTGTCCGAGCCATATGGTGA |
| *Ccl6* | TCAAGCCGGGCATCATCTTT | CTGCCCTCCTTCTCAAGCAA |
| *Ccl7* | GTCTGCCAGCTCTCACTGAA | GCATTGGGCCCATCTGGTTG |
| *Ccl8* | GACGCTAGCCTTCACTCCAA | GAGCCTTATCTGGCCCAGTC |
| *Ccl9* | CAGGCCGGGCATCATCTTTA | AGTAGCTGGCAGTTCACACC |
| *Cxcl1* | ACCGAAGTCATAGCCACACTC | CTCCGTTACTTGGGGACACC |
| *Cxcl2* | CCAGACAGAAGTCATAGCCACT | GGTTCTTCCGTTGAGGGACA |
| *Cxcl3* | CCCAGACAGAAGTCATAGCCA | GTGAGGGGCTTCCTCCTTTC |
| *Cxcl5* | GCCCCTTCCTCAGTCATAGC | AGCTTTCTTTTTGTCACTGCCC |
| *Cxcl7* | TGGGCTTCAGACTCAGACCT | ATGGGTCCATGCCATCAGATTT |
| *Cxcl10* | CCACGTGTTGAGATCATTGCC | GAGGCTCTCTGCTGTCCATC |
| *Cxcl11* | CCACAGCTGCTCAAGGCTTC | AACTTTGTCGCAGCCGTTAC |
| *Cxcl14* | GTGGACGGGTCCAAGTGTAA | CCTCGCAGTGTGGGTACTTT |
| *Csf2* | CAGGGTCTACGGGGCAATTT | CACAGTCCGTTTCCGGAGTT |
| *Csf1* | CTCTAGCCGAGGCCATGTG | CGCCCCACAGAAGAATCCAA |
| *Il4* | AAAATCACTTGAGAGAGATCATCGG | GTTGCTGTGAGGACGTTTGG |
| *Il10* | GGGGCCAGTACAGCCGGGAA | CTGGCTGAAGGCAGTCCGCA |
| *Il12* | TGGTTTGCCATCGTTTTGCTG | ACAGGTGAGGTTCACTGTTTCT |
| *Il1rn* | GCGGACGATCACTCCTTCTG | AGCCCCACATATTTGAAATTCCA |
| *Tnfa* | CGGTGCCTATGTCTCAGCCT | GAGGGTCTGGGCCATAGAAC |
| *Il1b* | GAAATGCCACCTTTTGACAGTG | TGGATGCTCTCATCAGGACAG |
| *Il6* | AGTTGCCTTCTTGGGACTGA | TCCACGATTTCCCAGAGAAC |
| *Il17a* | TTTAACTCCCTTGGCGCAAAA | CTTTCCCTCCGCATTGACAC |
| *Actb* | AACAGTCCGCCTAGAAGCAC | CGTTGACATCCGTAAAGACC |
